# Leukemia inhibitory factor drives glucose metabolic reprogramming to promote breast tumorigenesis

**DOI:** 10.1038/s41419-022-04820-x

**Published:** 2022-04-19

**Authors:** Xuetian Yue, Jianming Wang, Chun-yuan Chang, Juan Liu, Xue Yang, Fan Zhou, Xia Qiu, Vrushank Bhatt, Jessie Yanxiang Guo, Xiaoyang Su, Lanjing Zhang, Zhaohui Feng, Wenwei Hu

**Affiliations:** 1 Department of Radiation Oncology, Rutgers Cancer Institute of New Jersey, Rutgers University, New Brunswick, NJ USA; 2 Rutgers Cancer Institute of New Jersey, Rutgers University, New Brunswick, NJ USA; 3 Department of Medicine, Rutgers Robert Wood Johnson Medical School, Rutgers University, New Brunswick, NJ USA; 4 Department of Chemical Biology, Rutgers Ernest Mario School of Pharmacy, Piscataway, NJ USA; 5 Metabolomics Shared Resource, Rutgers Cancer Institute of New Jersey, Rutgers University, New Brunswick, NJ USA; 6 Department of Pathology, Princeton Medical Center, Plainsboro, NJ USA; 7 Department of Biological Sciences, Rutgers University, Newark, NJ USA; 8 Department of Pharmacology, Rutgers University, Piscataway, NJ USA

**Keywords:** Breast cancer, Cancer metabolism, Oncogenes

## Abstract

LIF, a multifunctional cytokine, is frequently overexpressed in many types of solid tumors, including breast cancer, and plays an important role in promoting tumorigenesis. Currently, how LIF promotes tumorigenesis is not well-understood. Metabolic reprogramming is a hallmark of cancer cells and a key contributor to cancer progression. However, the role of LIF in cancer metabolic reprogramming is unclear. In this study, we found that LIF increases glucose uptake and drives glycolysis, contributing to breast tumorigenesis. Blocking glucose uptake largely abolishes the promoting effect of LIF on breast tumorigenesis. Mechanistically, LIF overexpression enhances glucose uptake *via* activating the AKT/GLUT1 axis to promote glycolysis. Blocking the AKT signaling by shRNA or its inhibitors greatly inhibits glycolysis driven by LIF and largely abolishes the promoting effect of LIF on breast tumorigenesis. These results demonstrate an important role of LIF overexpression in glucose metabolism reprogramming in breast cancers, which contributes to breast tumorigenesis. This study also reveals an important mechanism underlying metabolic reprogramming of breast cancers, and identifies LIF and its downstream signaling as potential therapeutic targets for breast cancers, especially those with LIF overexpression.

## Introduction

As a multifunctional cytokine, leukemia inhibitory factor (LIF) regulates many important physiological and pathological processes, including embryonic implantation, pluripotency of embryonic stem cells, and the function of somatic stem cells [1–4]. Our previous studies have shown that LIF is transcriptionally regulated by tumor suppressor p53, and mediates p53 function in embryonic implantation [5, 6]. LIF has a complex role in tumorigenesis. LIF was originally identified as a factor that inhibits leukemia (therefore named as leukemia inhibitory factor) [7]. Recent studies [8–15], including ours [8–10, 14], have shown that LIF promotes the tumorigenesis of many types of solid tumors, including breast cancer. LIF is frequently overexpressed in different types of cancers, including breast cancer [8, 9, 11, 12]. LIF overexpression promotes proliferation, metastasis and chemo-resistance of cancer cells [8–11], and is associated with poor prognosis in various types of cancers [8, 9, 11]. LIF functions mainly through binding to its receptor complex composed of LIF receptor (LIFR) and glycoprotein 130 (gp130) to regulate different signaling pathways in a highly cell/tissue type- and context-dependent manner [1–3]. Studies reported that LIF can activate several oncogenic signaling pathways (e.g., AKT, mTOR, and STAT3 pathways), as well as inhibit tumor suppressive function of p53 in cancer cells, which in turn contribute to the oncogenic activity of LIF in cancers [5, 8–11, 13, 14]. While these studies began to unravel a critical role of LIF in cancer, the molecular underpinnings of this cytokine in cancer, especially in breast cancer, are still far from clear.

Metabolic reprogramming is a hallmark of cancer cells and a key contributor to the progression of cancer, including breast cancer [16–19]. Glucose is one of the major sources to provide carbon and energy for the rapid proliferation of cancer cells. The majority of cancer cells display dramatically enhanced glucose uptake and lactate production compared with normal cells, known as the Warburg effect, which is a key metabolic change in cancer [16–18, 20]. Currently, the mechanism underlying the reprogramming of glucose metabolism in breast cancer is incompletely understood. Breast cancer is the most frequently diagnosed cancer among women in the USA. Advanced breast cancers lack effective therapeutic options. Our recent studies have shown that LIF is frequently overexpressed in breast cancers (~50-60%) across different genetic subtypes, and is associated with poor prognosis in breast cancer patients [9, 10]. LIF promotes proliferation, growth of subcutaneous (*s.c*.) breast xenograft tumors, and metastasis of breast cancer cells [9, 14]. These results strongly suggest a critical role of LIF in breast cancer. Currently, the precise role and mechanism of LIF in breast cancer is poorly understood, and the role of LIF in metabolic rewiring in breast cancer is still unclear.

In this study, we found that LIF is a novel and unique driver for glucose metabolic reprogramming in breast cancer. By quantifying metabolites using liquid chromatography/mass spectrometry (LC/MS) and confirming with complementary methods, we found that LIF reprograms glucose metabolism in different breast cancer cells both in vitro and in vivo. Further, blocking glucose metabolic reprogramming genetically or pharmacologically largely abolished the promoting effect of LIF on breast tumorigenesis. Results from this study suggest that glucose metabolic reprogramming driven by LIF is critical for breast tumorigenesis, which is therapeutically targetable for LIF-overexpressing breast cancer.

## Results

### LIF promotes glucose uptake and drives glucose metabolic reprogramming

**Fig. 1:**
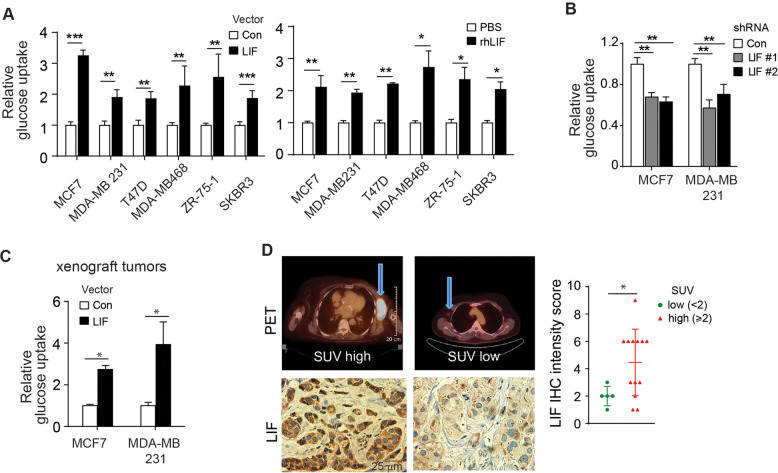
LIF promotes glucose uptake in breast cancer cells. **A** Ectopic LIF expression (left panel) as well as treatment with recombinant human LIF protein (rhLIF, 100 ng/ml for 6 h; right panel enhanced glucose uptake in breast cancer cell lines, including MCF7, MDA-MB 231, T47D, MDA-MB 468, ZR-75-1 and SKBR3 cells as determined by measuring the uptake of ^3^H-2-DG in cells. The overexpression of LIF in these cells was shown in Supplementary Fig 1A. **B** Knockdown of endogenous LIF by two different shRNAs reduced glucose uptake in MCF7 and MDA-MB 231 cells. The knockdown of LIF expression in these cells was shown in Supplementary Fig 1B. **C** Ectopic LIF expression increased glucose uptake in the xenograft tumors formed by MDF7 and MDA-MB 231 cells as determined by measuring the uptake of ^3^H-2-DG in tumor tissues. **D**. High LIF expression was associated with the increased ^18^F-FDG uptake in human breast tumors. Left panels: representative PET scan data and images of LIF IHC staining. SUV: standardized uptake value. In **A**–**C**, *n* = 3/group; in **D**, *n* = 18 for total patient samples. *: *p* < 0.05; **: *p* < 0.01; ***: *p* < 0.001; unpaired Student’s *t*-test for **A**–**C**; correlation in **D** was calculated by Spearman’s Rho correlation analysis.

Metabolic reprogramming is a hallmark of cancers. Glucose is a major source to provide carbon and energy for cancer cells to proliferate. Here, we investigated the effect of LIF on glucose uptake in different subtypes of human breast cancer cell lines. Human breast cancer cells MCF7 (luminal; estrogen receptor^+^ (ER^+^), progesterone receptor^+^ (PR^+^)), MDA-MB 231 (basal; triple-negative breast cancer, (TNBC)), T47D (luminal; ER^+^, PR^+^), MDA-MB468 (basal; TNBC), ZR-75-1 (luminal; ER^+^, PR^+^) and SK-BR3 (luminal; Her2^+^) were stably transduced with retroviral vectors expressing LIF, and the levels of glucose uptake were examined by measuring the uptake of ^3^H-2-deoxyglucose (^3^H-2-DG) as we previously described [21]. Ectopic LIF expression significantly increased glucose uptake in all of these breast cancer cells tested (Fig. 1A, left panel). LIF can function through both autocrine and paracrine manners. To determine whether the paracrine secretion of LIF has a similar effect on glucose uptake in breast cancer cells, above-mentioned cells were treated with recombinant human LIF protein (rhLIF). Treatment of rhLIF significantly increased glucose uptake in these cells (Fig. 1A, right panel). Results from our previous study showed that MCF7 and MDA-MB 231 cells have relatively high endogenous LIF expression compared with other breast cancer cells [10]. Therefore, we knocked down the endogenous LIF in these two cell lines by two different LIF shRNA retroviral vectors. Knockdown of the endogenous LIF significantly decreased glucose uptake in MCF7 and MDA-MB 231 cells (Fig. 1B). The effect of LIF on glucose uptake was further investigated in subcutaneous (*s.c*.) xenograft tumors formed by MCF7 and MDA-MB 231 cells *via* monitoring the uptake of ^3^H-2-DG by tumors as described previously [21]. Mice bearing tumors were injected (*i.p*.) with ^3^H-2-DG, and tumors were collected after 1.5 hours to measure the levels of ^3^H-2-DG in xenograft breast tumors. In both MCF7 and MDA-MB 231 xenograft tumors, ectopic LIF expression greatly increased glucose uptake in tumors (Fig. 1C). We further used the results from a group of breast cancer patients who received ^18^F-fluorodeoxyglucose (^18^F-FDG) microPET scans to evaluate the impact of LIF overexpression on glucose uptake in breast tumors of breast cancer patients in vivo. The LIF protein levels of breast tumor specimen from these patients were determined by immunohistochemistry (IHC) staining assays. Notably, the LIF protein level of these breast tumors was positively associated with glucose uptake reflected by the intensity of ^18^F-FDG PET scan in breast tumors (Fig. 1D).

**Fig. 2:**
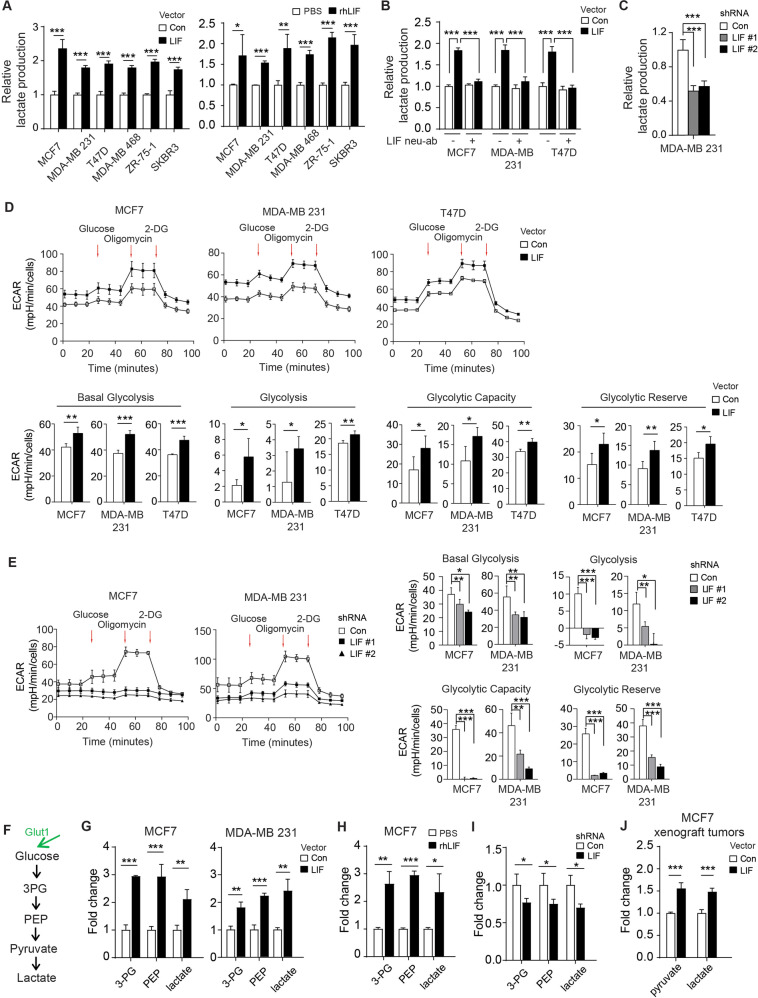
LIF drives glycolysis in breast cancer cells. **A** Ectopic LIF expression (left panel) or treatment with rhLIF (right) enhanced lactate production of different breast cancer cell lines analyzed by measuring lactate levels in the medium using a lactate assay kit. **B** LIF neutralization antibody (LIF neu-ab) blocked the promoting effect of LIF on lactate production in MCF7, MDA-MB 231 and T47D cells. **C** Knockdown of endogenous LIF decreased lactate production in MDA-MB 231 cells. **D**, **E** Ectopic LIF expression enhanced glycolysis rates (**D**), whereas knockdown of endogenous LIF decreased glycolysis rates (**E**) in different breast cancer cell lines as calculated by ECAR measured by using a Seahorse analyzer. **F** The diagram of glycolysis. **G**–**I** The fold change of inter-metabolites in glycolysis resulted from ectopic LIF expression in MCF7 and MDA-MB 231 cells (**G**), rhLIF treatment in MCF7 cells (**H**) or LIF knockdown in MDA-MB 231 cells (**I**) as determined by LC/MS metabolomics analysis. **J** The fold change of inter-metabolites in glycolysis induced by ectopic LIF expression in MCF7 xenograft tumors determined by LC/MS metabolomics analysis. Data are presented as mean ± SD (*n* = 3/group). *p < 0.05; **p < 0.01; ***p < 0.001; unpaired Student’s *t*-test.

The Warburg effect is characterized by the enhanced glucose uptake and lactate production in cancer cells. In addition to enhancing glucose uptake, both ectopic LIF expression and rhLIF treatments increased lactate production in the above-mentioned different breast cancer cell lines (Fig. 2A). The increased lactate production by LIF can be largely abolished by treating breast cancer cells with LIF neutralization antibody (LIF neu-ab) (Fig. 2B). Consistently, knockdown of LIF greatly decreased lactate production in MDA-MB 231 cells (Fig. 2C). Employing Seahorse Analyzer to measure extracellular acidification rate (ECAR) of cells as an indication of glycolytic activity, we found that ectopic LIF expression significantly increased glycolysis in MCF7, MDA-MB231, and T47D cells, while knockdown of LIF greatly decreased glycolysis in MCF7 and MDA-MB 231 cells (Fig. 2D, E).

To further define the role of LIF in glucose metabolism, we performed LC/MS-based metabolomics profiling in MCF7 and MDA-MB 231 cells with or without ectopic LIF expression. Figure 2F shows the diagram of glycolysis with intermediate metabolites. The analysis of metabolite pool size in cancer cells showed that LIF drove glucose metabolic reprogramming; ectopic LIF expression increased the levels of intermediates of glycolysis, including 3-phosphoglycerate (3-PG), phosphoenol pyruvate (PEP), and lactate (Fig. 2G). Similar observations were made in MCF7 cells treated with rhLIF (Fig. 2H). Consistently, LIF knockdown decreased the levels of these intermediate metabolites of glycolysis in MDA-MB 231 cells (Fig. 2I). We further compared the metabolite pool size between MCF7 xenograft tumors with and without ectopic LIF expression and obtained similar results showing the increased levels of pyruvate and lactate (Fig. 2J). Together, these data suggest that LIF promotes glucose uptake and drives glycolysis in breast cancer cells.

### The promoting effect of LIF on glucose uptake and glycolysis contributes to the tumor-promoting function of LIF

**Fig. 3:**
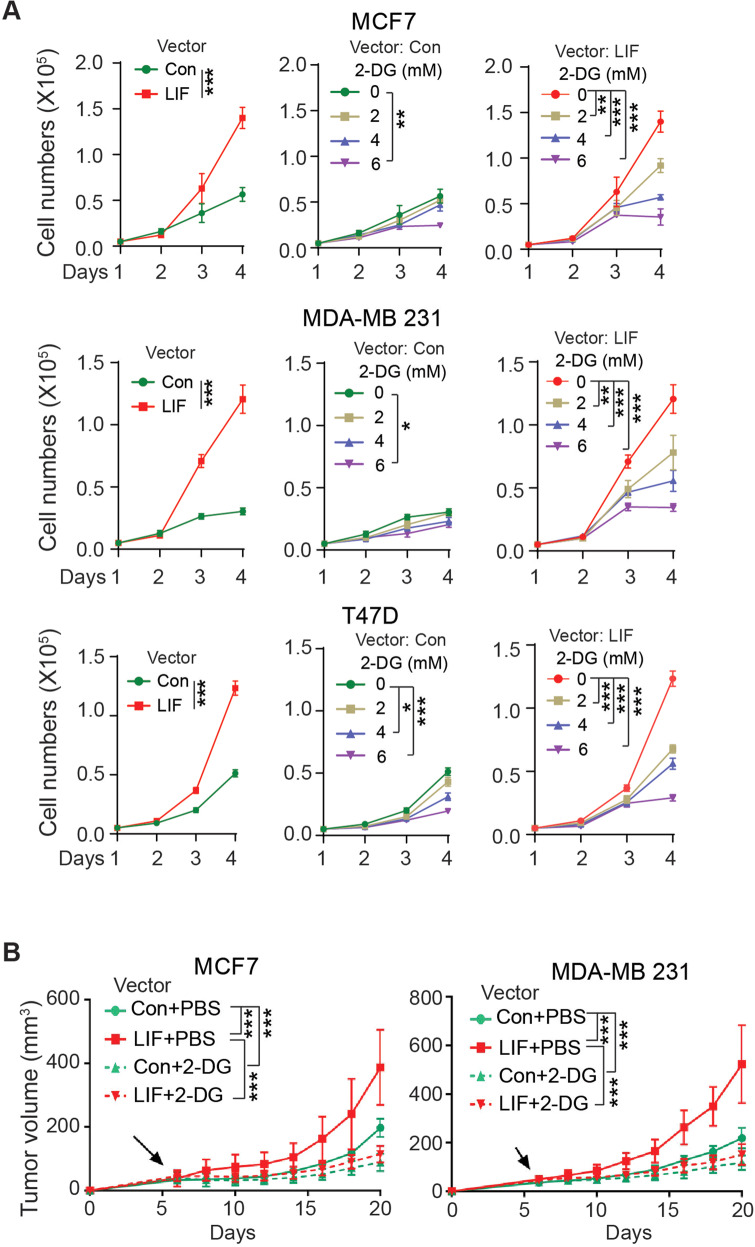
2-DG, a glycolysis inhibitor, preferentially inhibits the proliferation of breast cancer cells and the growth of xenograft tumors with LIF overexpression. **A** 2-DG preferentially inhibited the proliferation of breast cancer cells with ectopic LIF expression compared with control cells transduced with control vectors. MCF7, MDA-MB 231, and T47D cells with or without ectopic LIF expression were treated with 2-DG at different concentrations. **B** 2-DG treatment exhibited a more pronounced inhibitory effect on growth of xenograft tumors formed by MCF7 and MDA-MB 231 cells with ectopic LIF expression than those formed by control cells. Mice with xenograft tumors (~50 mm^3^) were administered with 2-DG (i.p., 1 g/kg mice, every two days for two weeks). Arrows represent the start of 2-DG treatment. Data are presented as mean ± SD. In **A**, *n* = 3/group; in **B**, *n* ≥ 5/group. *p < 0.05; **p < 0.01; ***p < 0.001; 2-way ANOVA followed by Student’s *t*-test.

It has been demonstrated that increased glucose uptake and glycolysis a key contributor to cancer cell proliferation and tumor progression [16–18]. Here, we investigated whether the promoting effect of LIF on glucose uptake and glycolysis contributes to the tumor-promoting function of LIF. Ectopic LIF expression significantly promoted the proliferation of MCF7, MDA-MB 231, and T47D cells (Fig. 3A). Glucose analog 2-DG competes with glucose and inhibits glucose transport and glycolysis [22]. 2-DG treatments inhibited the proliferation of MCF7, MDA-MB 231, and T47D cells in a dose-dependent manner (Fig. 3A). Notably, breast cancer cells with ectopic LIF expression were more sensitive toward 2-DG treatments than cells transduced with control vectors (Fig. 3B). We further compared the effect of 2-DG treatments on the growth of *s.c*. xenograft tumors formed by MCF7 and MDA-MB 231 cells with or without ectopic LIF expression. LIF overexpression promoted the growth of xenograft tumors formed by both MCF7 and MDA-MB 231 cells (Fig. 3B). For 2-DG treatments, mice with tumor volumes ~50 mm^3^ were administered with 2-DG (*p.o*., 1 g/kg mice, once every two days for two weeks). While 2-DG treatments inhibited the growth of xenograft tumors, a much more pronounced inhibitory effect of 2-DG was observed in tumors with LIF overexpression (Fig. 3B). Together, these results demonstrate that LIF promotes glucose uptake and glycolysis in breast cancer cells to promote cell proliferation and tumor growth.

### LIF induces plasma membrane (PM) translocation of glucose transporter 1 (Glut1) in breast cancer cells to enhance glycolysis and tumor growth

**Fig. 4:**
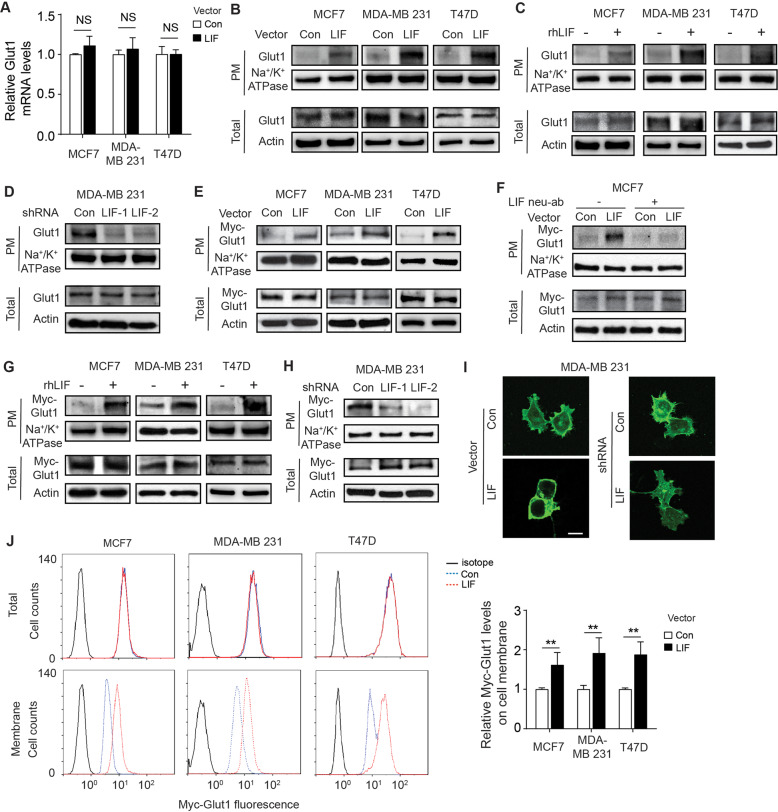
LIF induces Glut1 PM translocation in breast cancer cells. **A** Glut1 mRNA levels were detected by quantitative real-time PCR (qPCR) in MCF7, MDA-MB 231, and T47D cells with or without ectopic LIF expression. **B**, **C** Ectopic LIF expression (**B**) or rhLIF treatment (100 ng/ml for 12 h) (**C**) promoted endogenous Glut1 PM translocation in MCF7, MDA-MB 231, and T47D cells as determined by Western-blot assays. **D** Knockdown of LIF decreased endogenous Glut1 PM translocation in MDA-MB 231 cells. **E** Ectopic LIF expression increased the PM translocation of ectopically expressed Myc-Glut1 in MCF7, MDA-MB 231 and T47D cells as determined by Western-blot assays. **F** LIF neutralization antibody (LIF neu-ab) largely abolished exogenous Glut1 PM translocation promoted by LIF. **G** The rhLIF treatment promoted exogenous Glut1 PM translocation in cells. **H** Knockdown of LIF decreased Myc-Glut1 PM translocation in MDA-MB 231 cells. **I** Ectopic LIF expression promoted Myc-Glut1 PM translocation (left panels) while knockdown of LIF decreased Myc-Glut1 PM translocation (right panels) in MDA-MB 231 cells as determined by IF staining assays. Scale bar, 10 μm. **J** Ectopic LIF expression promoted the PM translocation of Myc-Glut1 in MCF7, MDA-MB 231, and T47D cells as determined by flow cytometry assays. Left panels: representative images of flow cytometry analysis. Right panels: quantifications of relative fluorescence intensity of Myc-Glut1 on the cell membrane normalized with total Myc-Glut1 fluorescence intensity in cells. In **A**, **J** data are presented as mean ± SD. *n* = 3/group. *p < 0.05; **p < 0.01; NS: non-significant; unpaired Student’s *t*-test. Uncropped Wes*t*ern-blot images are shown in Supplementary Fig 2.

Glucose transportation is the first rate-limiting step of glucose metabolism, which is controlled by glucose transporters (Gluts) [21, 23]. Among Gluts, Glut1 is widely expressed in almost all types of cells and tissues, and is responsible for their basal glucose uptake [24]. In addition to Glut1 overexpression, enhanced Glut1 translocation from the intracellular pool to the PM is frequently observed in different types of cancers, including breast cancer, which enhances glucose uptake in cancer [21, 25, 26]. We found that ectopic LIF expression had no obvious effect on Glut1 mRNA expression as determined by quantitative real-time PCR (qPCR) in MCF7, MDA-MB 231, and T47D cells (Fig. 4A). We further determined the levels of Glut1 on the PM in breast cancer cells by Western-blot analysis of the fraction of the PM isolated from cells, and found that ectopic LIF expression promoted endogenous Glut1 PM translocation but did not change total Glut1 protein levels in MCF7, MDA-MB 231 and T47D cells (Fig. 4B). Similar results were obtained when cells were treated with rhLIF (Fig. 4C). Furthermore, knockdown of LIF reduced PM translocation of endogenous Glut1 in MDA-MB 231 cells (Fig. 4D). The effect of LIF on Glut1 PM translocation was further examined in breast cancer cells transfected with vectors expressing Myc-Glut1. Ectopic LIF expression clearly promoted the PM translocation of Myc-Glut1 in MCF7, MDA-MB 231, and T47D cells, which can be blocked by LIF neu-ab, as determined by Western-blot assays (Fig. 4E, F). Similar results were obtained when these cells were treated with rhLIF (Fig. 4G). Further, knockdown of LIF reduced the PM translocation of Myc-Glut1 in MDA-MB 231 cells (Fig. 4H). The promoting effect of LIF on Myc-Glut1 PM translocation was also confirmed by immunofluorescence (IF) staining assays and flow cytometry assays in these cells (Fig. 4I, J).

**Fig. 5:**
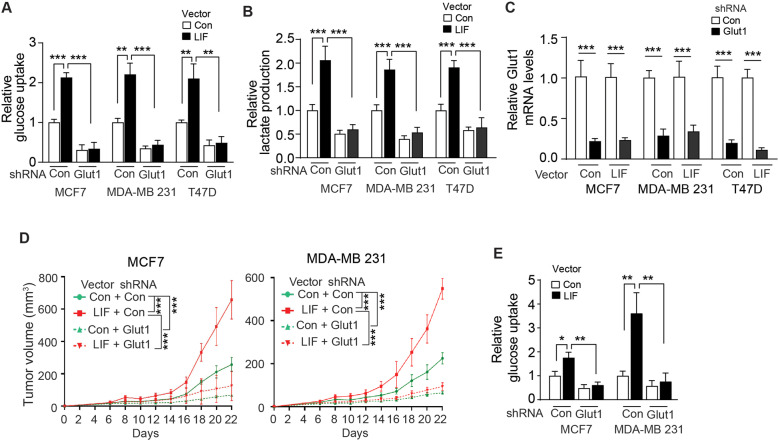
Knockdown of endogenous Glut1 largely abolishes enhanced glycolysis driven by LIF in breast cancer cells and the promoting effect of LIF on xenograft breast tumor growth. Endogenous Glut1 was knocked down by shRNA in MCF7, MDA-MB 231, and T47D cells with or without ectopic LIF expression. **A**, **B** Glucose uptake (**A**) and lactate production (**B**) were measured in cells. **C** The efficiency of Glut1 knockdown was determined at mRNA levels by qPCR. **D** Knockdown of Glut1 greatly inhibited the promoting effect of LIF on xenograft tumor growth. MCF7 and MDA-MB 231 cells with or without ectopic LIF expression along with or without Glut1 knockdown were employed for xenograft tumorigenesis assays. **E** Knockdown of Glut1 greatly blocked the promoting effect of LIF on glucose uptake in xenograft tumors formed by MCF7 and MDA-MB 231 cells. Glucose uptake was measured in xenograft tumors described in **D**. Data are presented as mean ± SD. In **A**, **B**, **C** & **E**: *n* = 3/group; in **D**: *n* = 6/group. *p < 0.05; **p < 0.01; ***p < 0.001; unpaired Student’s *t*-test for **A**, **B**, **C**, **E**, and 2-way ANOVA followed by Student’s *t*-test for D.

We then investigated whether Glut1 mediates the promoting effect of LIF on glucose uptake and glycolysis. Knockdown of endogenous Glut1 by retroviral shRNA vectors greatly reduced glucose uptake and lactate production in MCF7, MDA-MB 231 and T47D cells (Fig. 5A, B). Notably, knockdown of Glut1 largely abolished the promoting effect of LIF on glucose uptake and lactate production in these cells (Fig. 5A, B). The knockdown of Glut1 RNA levels by shRNA vectors in these cells was confirmed by qPCR assays (Fig. 5C).

We further investigated the effect of LIF overexpression on the growth of xenograft breast tumors with or without endogenous Glut1 knockdown. LIF overexpression promoted the growth of xenograft tumors formed by MCF7 and MDA-MB 231 cells (Fig. 5D). Knockdown of endogenous Glut1 greatly inhibited the growth of xenograft tumors formed by MCF7 and MDA-MB 231 cells. Notably, Glut1 knockdown largely abolished the promoting effect of LIF on the growth of xenograft tumors (Fig. 5D). LIF promotes glucose uptake of xenograft tumors formed by MCF7 and MDA-MB 231 cells (Fig. 5E). Knockdown of Glut1 greatly inhibited glucose uptake in MCF7 and MDA-MB 231 xenograft tumors, and largely abolished the promoting effect of LIF on glucose uptake (Fig. 5E). These results support that PM translocation of Glut1 promoted by LIF contributes greatly to the promoting effect of LIF on glucose uptake and breast tumorigenesis.

### LIF promotes Glut1 PM translocation and glycolysis through the AKT signaling pathway

**Fig. 6:**
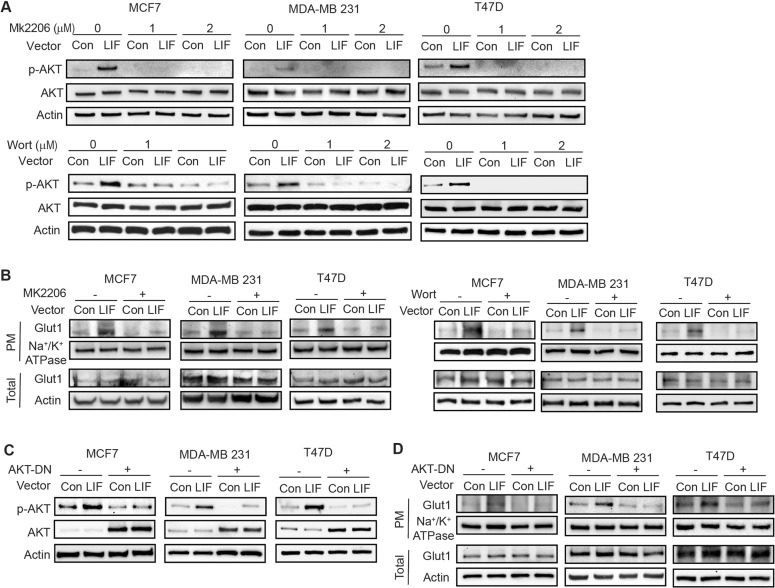
LIF promotes Glut1 PM translocation through the activation of AKT signaling. **A** LIF activated the AKT signaling, which can be blocked by MK2206, an AKT inhibitor (upper panel), or Wortmannin (Wort), a PI3K inhibitor (lower panel), in MCF7, MDA-MB 231 and T47D cells. The levels of p-AKT^Ser473^ (p-AKT), which reflect the activity of AKT were measured by Western-blot assays. **B** Blocking AKT signaling by MK2206 (left) or Wortmannin (right) largely abolished the promoting effect of LIF on Glut1 PM translocation in breast cancer cells. **C**, **D** Blocking AKT signaling by expression of dominant-negative AKT (AKT-DN) largely abolished the promoting effect of LIF on Glut1 PM translocation in breast cancer cells. Cells were transfected with vectors expressing AKT-DN. The levels of p-AKT and total AKT (**C**), as well as Glut1 PM translocation (**D**), were determined by Western-blot assays. Uncropped Western-blot images are shown in Supplementary Fig 3.

The AKT signaling pathway is frequently activated in different types of cancers [27]. AKT activation has been reported to promote the PM translocation of Glut1 to enhance glycolysis [28]. Previous reports, including ours, showed that LIF activates AKT in cancer cells [4, 9], which raises the possibility that AKT activation by LIF contributes to the promoting effect of LIF on Glut1 PM translocation. To this end, MCF7, MDA-MB 231 and T47D cells were treated with MK2206 [29], a specific small molecule AKT inhibitor, or Wortmannin [30], a PI3K/AKT inhibitor, to test whether AKT mediates the promoting effect of LIF on Glut1 PM translocation. Ectopic LIF expression activated AKT, which was reflected by the increased phosphorylation levels of AKT at Serine 473 (p-AKT) in MCF7, MDA-MB 231 and T47D cells (Fig. 6A). AKT activation by LIF was largely blocked by MK2206 and Wortmannin treatments in these cells (Fig. 6A). Notably, MK2206 and Wortmannin treatments greatly reduced Glut1 PM translocation promoted by LIF but did not affect the total Glut1 protein levels in these cells (Fig. 6B). Consistent results were obtained when cells were transfected with a vector expressing dominant negative AKT (AKT-DN) to block the AKT signaling in cells (Fig. 6C); expression of AKT-DN largely blocked the Glut1 PM translocation driven by LIF but did not affect the total Glut1 protein levels in MCF7, MD-MB 231 and T47D cells (Fig. 6D).

**Fig. 7:**
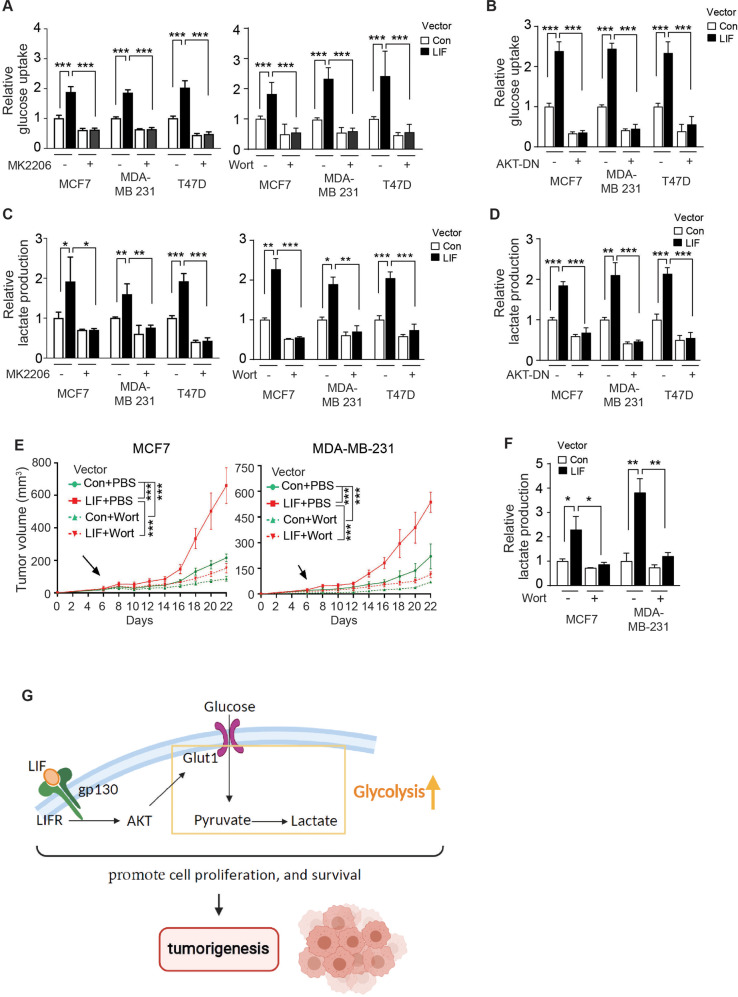
Blocking ATK activation largely abolishes glycolysis driven by LIF in breast cancer cells and the promoting effect of LIF on xenograft breast tumor growth. **A**, **B** Blocking AKT activation by MK2206 (1 µM for 12 hours) (**A**, left panel), Wortmannin (2 µM for 6 h) (**A**, right panel) or AKT-DN expression (**B**) largely abolished the promoting effect of LIF on glucose uptake in breast cancer cell lines. **C**, **D**. Treatment with MK2206 (**C**, left panel), Wortmannin (**C**, right panel) or AKT-DN expression (**D**) largely abolished the promoting effect of LIF on lactate production in breast cancer cell lines. **E**. Blocking AKT signaling by Wortmannin greatly inhibited the promoting effect of LIF on xenograft tumor growth. Mice with xenograft tumors (~50 mm^3^) were administered with Wortmannin (*i.p*., 1.5 mg/kg mice, daily for two weeks). **F** Wortmannin treatment largely abolished the promoting effect of LIF on glucose uptake in xenograft tumors. Glucose uptake was measured in xenograft tumors described in **E**. **G** Schematic illustration of the role of LIF in increasing glycolysis, which in turn promotes breast tumorigenesis. Data are present as mean ± SD. For **A**–**D**, **F**: *n* = 3/group; For **E**: *n* ≥ 6/group. *p < 0.05; **p < 0.01; ***p < 0.001; unpaired Student’s *t*-test for **A**–**D**, **F**, and 2-way ANOVA followed by Student’s *t*-test for **E**.

Our results further showed that blocking the AKT signaling by either treatments of MK2206 and Wortmannin or expression of AKT-DN greatly reduced glucose uptake (Fig. 7A, B) and lactate production (Fig. 7C, D), and largely abolished the increased glucose uptake (Fig. 7A, B) and lactate production (Fig. 7C, D) promoted by LIF in breast cancer cells. These results support that AKT activation by LIF is an important mechanism for the enhanced glycolysis driven by LIF in breast cancer cells.

We further tested whether Wortmannin treatment blocks the promoting effect of LIF on the growth of xenograft breast tumors. Wortmannin was used to treat mice (1.5 mg/kg body weight/day for 16 days) when tumor volume reached ~50 mm^3^. Wortmannin significantly inhibited the growth of MCF7 and MDA-MB 231 xenograft tumors. Notably, the inhibitory effect of Wortmannin treatment was much more pronounced in tumors with ectopic LIF overexpression than tumors formed by control cells transduced with a control vector (Fig. 7E). Further, Wortmannin treatment largely abolished the promoting effect of LIF on glucose uptake in MCF7 and MDA-MB 231 xenograft tumors (Fig. 7F). Taken together, these results strongly suggest that AKT activation by LIF is an important mechanism whereby LIF promotes glycolysis and breast tumorigenesis (Fig. 7G).

## Discussion

Metabolic reprogramming is a cancer hallmark. Aberrant activation of oncogenic pathways and/or inactivation of tumor suppressive pathways, including p53, Myc, Ras and HIF, play critical roles in driving metabolic reprogramming in cancer [16, 17, 31]. LIF displays a variety of functions in a highly cell-, tissue- and context-dependent manner in many physiological and pathological processes, including regulating cell proliferation, stem cell functions, embryonic implantation, and inflammation [1–4]. Recent studies also suggest that LIF plays a critical role in tumorigenesis. LIF is frequently overexpressed in many cancer types, including breast cancer, which promotes proliferation, metastasis and therapeutic resistance of cancer cells [8, 9, 11, 12]. In clinic, LIF overexpression often correlates with poor prognosis in many cancer types, including breast cancer [8, 9, 11, 15, 32]. Currently, the underlying mechanisms whereby LIF promotes tumorigenesis are still not well understood, and the role of LIF in cancer metabolic reprogramming is unclear. In this study, we found that LIF drives glycolysis, contributing to breast tumorigenesis (Fig. 7G).

The aerobic glycolysis (the Warburg effect) is the most well-characterized metabolic change in cancer cells; the majority of cancer cells exhibit enhanced utilization of aerobic glycolysis even under normal oxygen concentrations, and display dramatically enhanced glucose uptake and lactate production compared with normal cells [17, 33–35]. This study demonstrates that elevated LIF expression in breast cancer cells promoted glycolysis with significantly increased glucose uptake, lactate production, and glycolytic rate. This upregulation and reprogramming of glucose metabolism induced by LIF overexpression were observed in different subtypes of breast cancers, including luminal A, basal-like, Her2+, and TNBC cancers, in both in vitro cultured cells and in vivo xenograft tumor models. Notably, high LIF levels are also associated with increased glucose uptake in human breast tumor tissues. Our results further showed that blocking glucose uptake by 2-DG inhibited the proliferation of breast cancer cells and growth of xenograft breast tumors, and largely abolished the promoting effect of LIF on the proliferation of breast cancer cells and growth of xenograft breast tumors. These results indicate that the rewiring of glucose metabolism contributes to the promoting effect of LIF on breast tumorigenesis.

Results from this study further show that AKT activation by LIF is an important mechanism by which LIF rewires glucose metabolism in breast cancer cells. LIF overexpression in the breast cancer cells activated the AKT signaling to promote Glut1 PM translocation, which in turn led to enhanced glucose uptake and glycolysis. Knockdown of Glut1 or blocking AKT signaling by inhibitors largely abolished the promoting effect of LIF on glucose uptake, glycolysis as well as its promoting effect on the growth of xenograft breast tumors.

As a cytokine, LIF functions through both autocrine and paracrine manners. In this study, we observed that both LIF overexpressed in breast cancer cells and recombinant LIF protein added into the culture medium rewired glucose metabolism in breast cancer cells. Currently, it is unclear the role of LIF in metabolic reprogramming in different cell types in the tumor microenvironment, including tumor-associated fibroblasts and immune cells. Considering that LIF functions in a highly cell- and tissue type-specific manner, future studies are needed to elucidate the impact of LIF overexpression upon different cell types in the tumor microenvironment. It has been reported that LIF is frequently overexpressed in many cancer types, including pancreatic cancer, colon cancer, and prostate cancer [8, 9, 11, 12]. It will be interesting to elucidate whether LIF plays a similar role in glucose metabolic rewiring in those cancer types, which in turn contributes to tumorigenesis.

In summary, this study demonstrates an important role of LIF overexpression in glucose metabolism reprogramming in breast cancers, which contributes to breast tumorigenesis. LIF overexpression enhances glucose uptake and glycolysis via the AKT/Glut1 axis. Given that LIF is frequently overexpressed in breast cancer, results from this study reveal an important mechanism underlying metabolic reprogramming of breast cancer, and identify LIF and its relevant downstream signaling as potential therapeutic targets for breast cancer, especially those with LIF overexpression.

## Methods and materials

### Cells, shRNA vectors, expression vectors, and chemical compounds

Breast cancer cell lines MCF7, MDA-MB 231, T47D, ZR-75-1, and MDA-MB 468 were purchased from ATCC and cultured in DMEM or RPMI1640 supplemented with 10% fetal bovine serum. The cell lines with stable ectopic LIF expression were established, as previously described [9]. All cell lines were authenticated by short tandem repeat profiling. Cells were regularly tested for mycoplasma using Lookout Mycoplasma PCR detection kit (MP0035, Sigma) and only used when negative. shRNA vectors targeting human LIF (SHCLNDNM_002309, Sigma) or human Glut1 (V3LHS_321625 and V3LHS_321626, Open Biosystems) were validated as we previously described [9, 21]. The LIF expression vector was established as previously described [9]. Briefly, the LIF cDNA fragment was amplified by PCR with following primer pairs: 5′AAGCTTATGAAGGTCTTGGCGGCAGGAG-3′; 5′-TGAATTCGCGAAGGCCTGGGCCAA-3′. The fragment was inserted into p3XFlag-CMV-14 vectors, then subcloned into pLPCX vectors along with the flag tag. The pLHCX-DN-AKT vector expressing a dominant negative AKT (DN-AKT; K179M) was constructed by subcloning the DN-AKT fragment from pLNCX-AKT1 K179M (Addgene) into the pLHCX vectors. Wortmannin and 2-DG were purchased from Sigma. MK-2206 dihydrochloride was purchased from MedChemExpress. ^3^H-2-DG was purchased from American Radiolabeled Chemicals. LIF neutralization antibody (AF-250-NA) was purchased from R&D Systems.

### FDG-PET/CT acquisition and IHC assays

De-identified primary breast tumor tissue samples and matched FDG-PET imaging on tumor samples were obtained from Princeton Cancer Tissue Repository with an IRB approval from Princeton Medical Center. IHC staining was performed as previously described [8]. Antibody against LIF (anti-LIF; 39N7D10, Novus, 1:500) was used for IHC staining.

### Lactate measurement

Cells were cultured in phenol red-free medium for 24 h. Lactate levels in the culture medium were measured using a lactate assay kit (K607, BioVision) according to the manufacture’s instruction, and normalized to cell numbers.

### Glucose uptake assays

Glucose uptake in cells was measured as previously described [21]. Briefly, cells cultured in 12-well plates were pre-incubated in glucose-free medium for 30 min and then cultured for 30 min in medium containing ^3^H-2-DG (1 µCi/well). Cells were lysed in 1% SDS. The radioactivity of cell lysates was measured by a liquid scintillation counter, and normalized to the protein concentrations of cell lysates.

Assays for glucose uptake in xenograft tumors were performed as previously described [21]. Briefly, mice bearing xenograft tumors were injected (*i.p*.) with ^3^H-2-DG (1 µCi/g body weight) and xenograft tumors were collected at 1.5 h after injection to measure the levels of ^3^H-2-DG-6-phosphate accumulated in tumors. All mouse experiments were approved by the Institutional Animal Care and Use Committee of Rutgers University.

### Cell proliferation assays

Cells were seeded on 24-well plates overnight, then treated with different concentrations of 2-DG. Cell numbers were analyzed by Vi-Cell Counter (Beckman) every day for 3 days.

### Xenograft breast tumorigenesis assays

MCF7 and MDA-MB 231 cells were injected (*s.c*.) into the 6-week-old BALB/c female nude mice (Taconic) for xenograft tumor formation. The nude mice were implanted a 17β-estradiol pellet (Innovative Research of America) 2 days before cell injection. When tumor volumes reached ~50 mm^3^, mice were treated with 2-DG (1 g/kg mouse body weight, every two days for two weeks), Wortmannin (1.5 mg/kg mouse body weight, daily for 16 days) or vehicle control. Tumors volume was monitored every two days. Tumor volume was calculated as following: tumor volume = ½ (L x W^2^), where L is the longitudinal diameter and W is the transverse diameter. All mouse experiments were approved by the Institutional Animal Care and Use Committee of Rutgers University.

### Metabolomics profiling by LC/MS assays

Total soluble metabolites from cells and tumors were analyzed by LC/MS assays as previously described [36]. Briefly, cells cultured in DMEM for 24 h were quickly washed with ice-cold PBS and metabolites were extracted in 1 ml of −80 °C methanol: acetonitrile: water (v/v = 40/40/20) with 0.5% formic acid for LC/MS analysis. The levels of metabolites were normalized with total cell numbers. For xenograft tumors, 30 mg xenograft tissues were treated with 0.8 ml of Methanol: Acetonitrile: H_2_O (40:40:20) with 0.5% Formic acid for 3 min on ice, then added with 13.3 µl of 15% NH_4_HCO_3_ and the tube was vortexed briefly to extract soluble metabolites for LC/MS analysis.

### Western-blot assays

The standard protocol was used to extract proteins and perform Western-blot assays as previously described [37]. Antibodies against Glut1 (ab14683, Abcam; 1:1,000), anti-LIF (AF-250-NA, R & D; 1:1000), anti-Na/K ATPase (ab58479, Abcam, 1:2,000), anti-p-AKT (4051, Cell Signaling; 1:2,000), anti-AKT (SC-1618, Santa Cruz; 1:2,000), and anti-β-actin (A5441, Sigma; 1:125,000) were used in this study.

### Quantitative real-time PCR assays

Total RNA was freshly extracted from 5 × 10^5^ cells using the RNeasy miniKit (Qiagen, 74106), and eluted in 50-µl RNase-free H_2_O. Samples with A260/A280 ratio beyond 1.8–2.1 were excluded from further experiments. Reverse transcription was then performed using 1000 ng total RNA in a 50-µL reaction system with the TaqMan Reverse Transcription Reagents (Applied biosystems, N8080234) and followed manufacturer’s operating protocol for reaction conditions. The Taqman primers for human LIF (Hs00171455_m1) and human actin (Hs99999903_m1) were purchased from Applied Biosystems. The Taqman assays were performed using Taqman^TM^ Gene Expression Master Mix (Applied biosystems, 4369016) following manufacturer’s operating instruction. The expression levels of Glut1 were determined by the SYBR Green assays. Sequences of the SYBR Green primers are as following: Glut1 Forward: TTGCAGGCTTCTCCAACTGGAC, Reverse: CAGAACCAGGAGCACAGTGAAG, Actin Forward: CACCATTGGCAATGAGCGGTTC, Reverse: AGGTCTTTGCGGATGTCCACGT. The SYBR Green assays were performed using PowerUp^TM^ SYBR Green Master Mix (Applied biosystems, A25778) following manufacturer’s operating instruction. Both Taqman and SYBR Green assays were performed and analyzed using the StepOnePlus^TM^ Real-Time PCR System (Applied biosystems, 4376600) following manufacturer’s instruction. The mRNA expression levels of genes were normalized with the β-actin gene.

### ECAR and glycolysis rate

ECAR and glycolysis rate were measured by using a Seahorse Biosciences extracellular flux analyzer (XF24), as previously described [38]. Briefly, cells were seeded at 2.5 × 10^4^ to 3 × 10^4^ cells in the XF24 plates overnight, and then subjected to XF assays according to the manufacturer’s instruction. The basal glycolysis, glycolysis, glycolytic capacity, and glycolytic reserve were determined as described previously [39].

### Membrane protein extraction

The PM fraction of cells was isolated and separated from the other membrane fraction of cells using the Qproteome Cell Compartment Kit (Qiagen) according to the manufacturer’s instruction. The expression levels of GLUT1 in the PM fraction were measured by Western-blot assays. A PM protein Na^+^/K^+^ ATPase was detected as an internal standard.

### Flow cytometry assays for Glut1 PM translocation

The PM traslocation of Glut1 was analyzed by flow cytometry assays as previous described [21]. Briefly, cells were transduced with lenti-viral vectors expressing Myc-Glut1 (a gift from Dr. Jeffrey Pessin at Albert Einstein College of Medicine). Glut1 levels on the cell membrane were examined at 48 hours after infection. Cells were blocked in PBS with 2% fetal bovine serum, and then stained with a Myc antibody (Roche) for the flow cytometry analysis. To determine total Glut1 levels, cells were fixed with 2% paraformaldehyde and permeabilized with 0.5% Triton X-100, and then stained with a Myc antibody for the flow cytometry analysis.

### IF staining assays

IF staining assays of cells were performed as described previously [21]. Briefly, cells cultured on coverslips were washed with ice-cold PBS and fixed with methanol. After the permeability treatment with PBS containing 0.1% Triton X-100, the coverslips were incubated with the anti-myc antibody (9E10, Roche; 1:100) overnight followed by Alexa Fluor 488-conjugated goat secondary antibody (Invitrogen; 1:200) for 1 h. The coverslips were mounted in Vectashield (Vector Laboratories) and examined by confocal laser-scanning microscopy.

### Statistical analysis

Sample sizes were chosen based on the power calculation. Samples/animals were randomly assigned to different treatment groups. The investigators were blinded to the group allocation during experiments and when assessing outcomes. Statistical analysis was performed by using the GraphPad Prism software. The significant differences in cell proliferation and the growth rate of xenograft tumors among groups were analyzed by ANOVA followed by Student’s *t*-tests using a GraphPad Prism software. All other *P* values were obtained using unpaired Student’s *t*-tests. Normal distribution of data in each group was tested by Shapiro-Wilk test. *p* < 0.05 was considered as statistically significant.

## Supplementary information

Supplementary figure

Reproducibility checklist

## Data Availability

All data needed to evaluate the conclusions in the paper are present in the paper and are available from the corresponding author upon reasonable request.
