# Supplementary material for: Leukemia inhibitory factor drives glucose metabolic reprogramming to promote breast tumorigenesis
Source: Cell Death Dis. 2022 Apr 19;13(4):370. doi: 10.1038/s41419-022-04820-x (PMC9018736; doi:10.1038/s41419-022-04820-x)
Supplement: Supplementary file 1 — Supplementary figure [file 41419_2022_4820_MOESM1_ESM.pdf]

## Supplementary figure

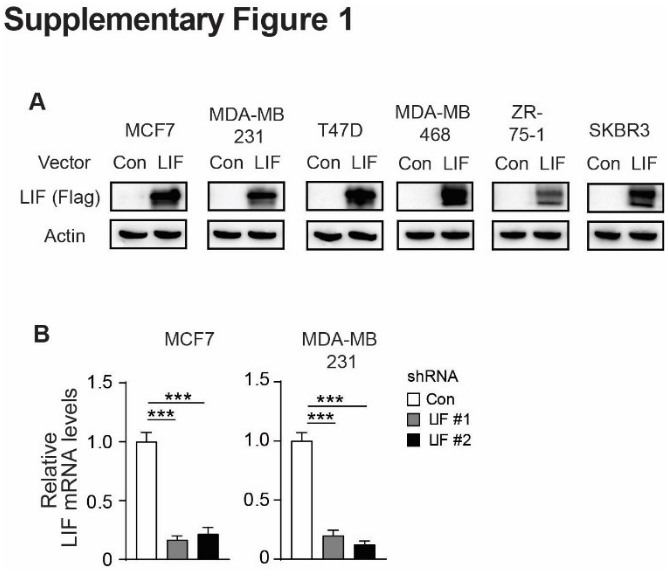


**Supplementary Figure 1. LIF expression levels in breast cancer cells with ectopic LIF expression or knockdown of endogenous LIF. A.** LIF protein levels in breast cancer cells with ectopic LIF expression determined by Western-blot assays. **B.** LIF mRNA levels in breast cancer cells with knockdown of endogenous LIF determined by quantitative real-time PCR. Uncropped Western-blot images are shown in **Supplementary Figure 4**.

## Supplementary Figure 2

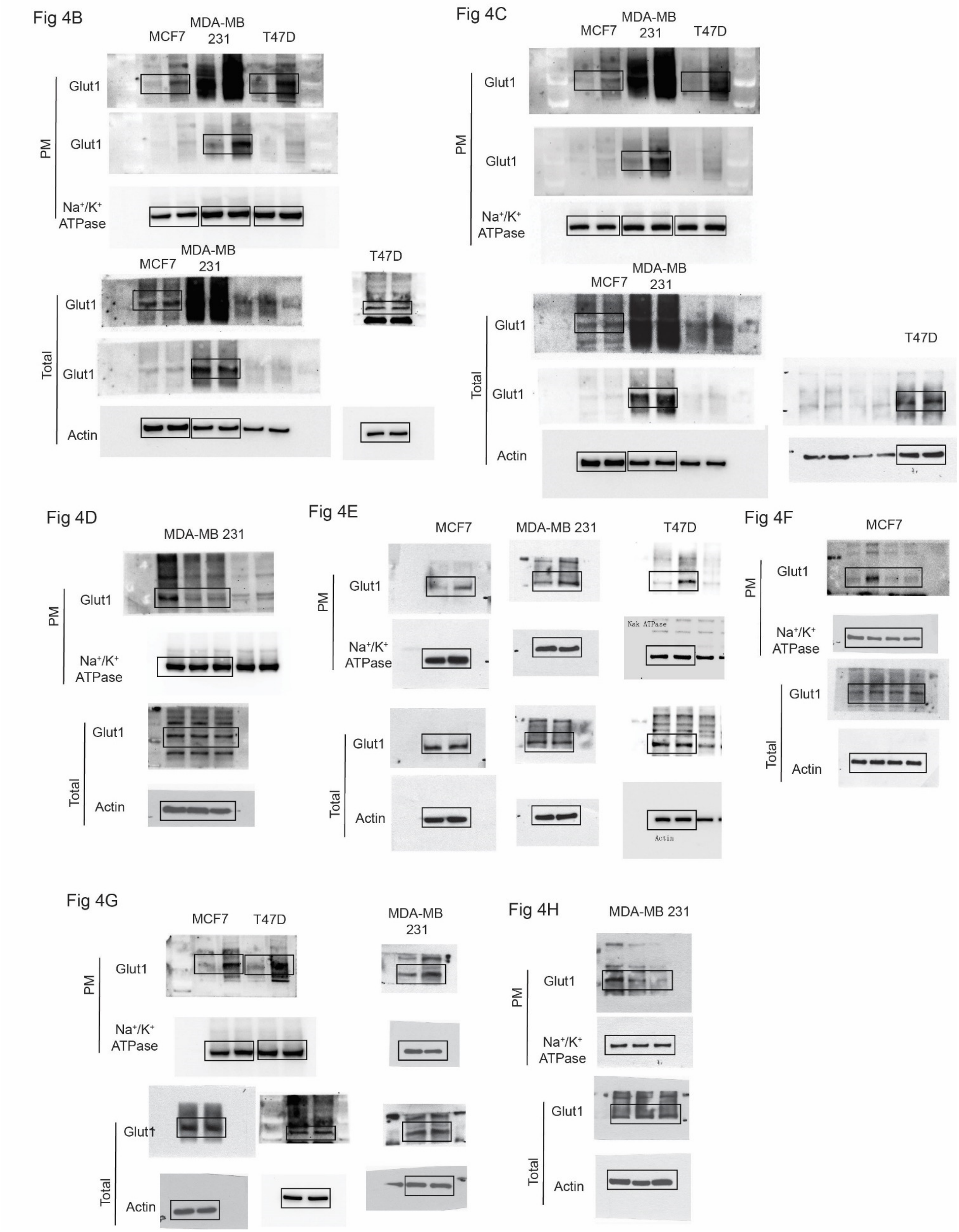


**Supplementary Figure 2. Uncropped Western-blot images for panels in Figure 4.**

## Supplementary Figure 3

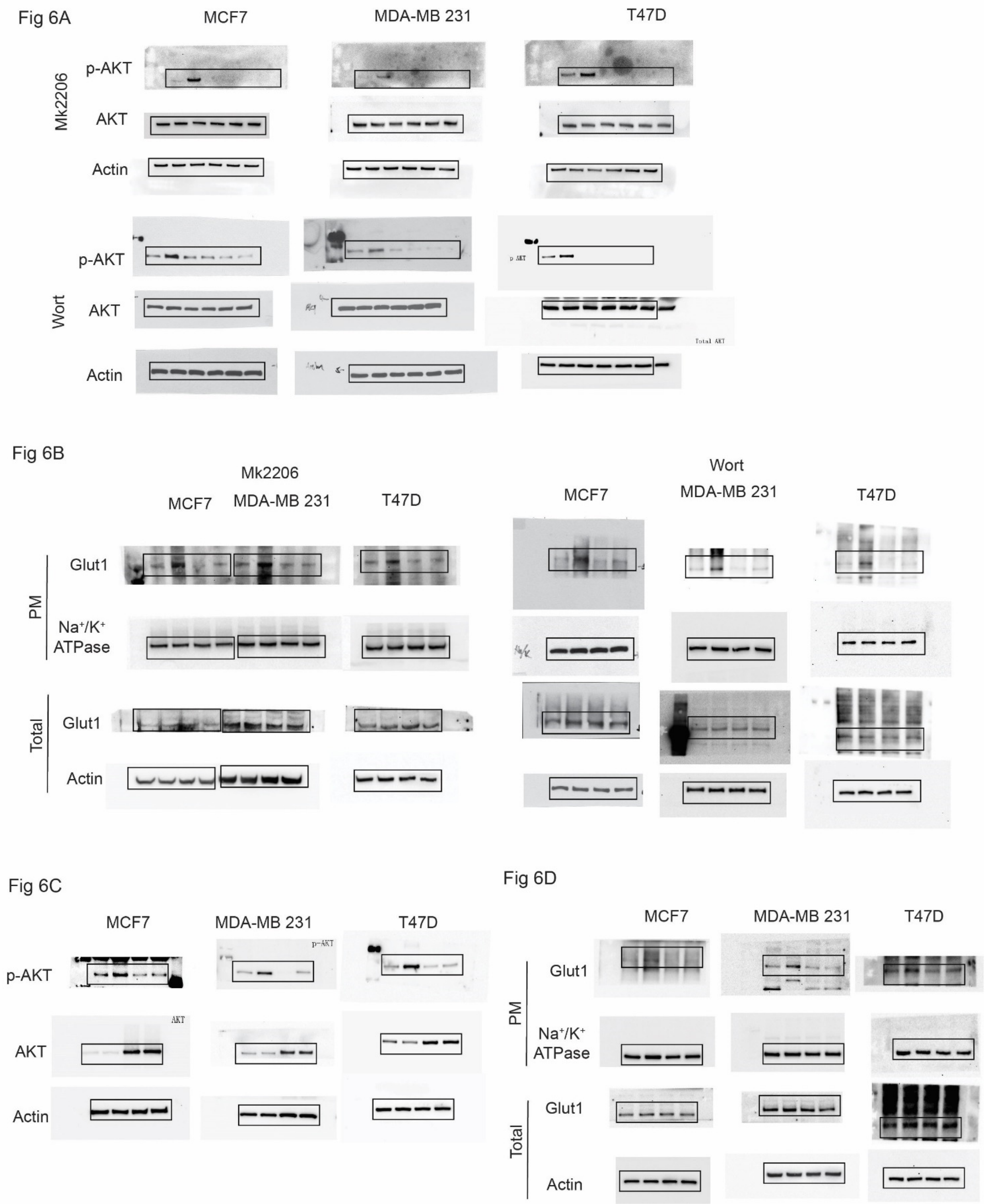


**Supplementary Figure 3. Uncropped Western-blot images for panels in Figure 6.**

## Supplementary Figure 4

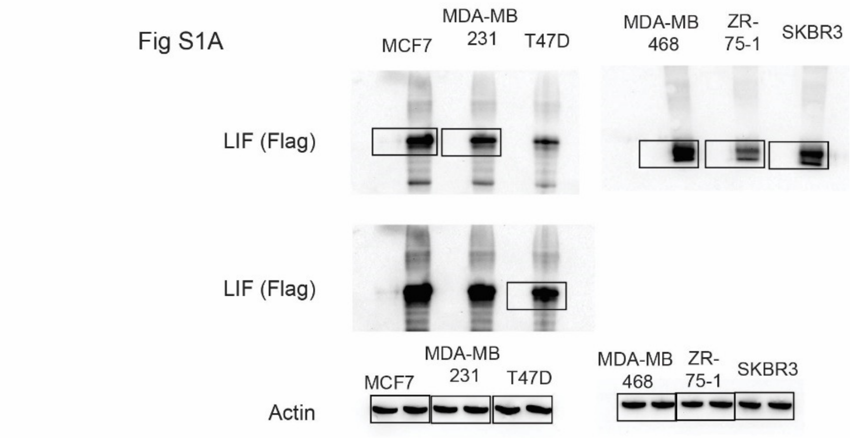


**Supplementary Figure 4. Uncropped Western-blot images for panels in Supplementary Figure 1.**
